# Cardiovascular Health Metrics in the Development and Regression of Nonalcoholic Fatty Liver Disease: A Cohort Study

**DOI:** 10.3390/jcm8050610

**Published:** 2019-05-06

**Authors:** Eun-Hee Jang, Yoosoo Chang, Seungho Ryu, Seolhye Kim, Young Hwan Kim, Ki-Chul Sung, Yong Kyun Cho, Soo-Jin Lee, Hocheol Shin, Sarah H. Wild, Christopher D. Byrne

**Affiliations:** 1 Center for Cohort Studies, Total Healthcare Center, Kangbuk Samsung Hospital, Sungkyunkwan University School of Medicine, Seoul 04514, Korea; eunhee2626.jang@samsung.com (E.-H.J.); seolhye.kim@samsung.com (S.K.); hcfm.shin@samsung.com (H.S.); 2 Department of Occupational and Environmental Medicine, Kangbuk Samsung Hospital, Sungkyunkwan University School of Medicine, Seoul 03181, Korea; 3 Department of Clinical Research Design & Evaluation, SAIHST, Sungkyunkwan University, Seoul 06351, Korea; 4 Department of Nuclear Medicine, Kangbuk Samsung Hospital, Sungkyunkwan University School of Medicine, Seoul 03181, Korea; yh27.kim@samsung.com; 5 Division of Cardiology, Department of Internal Medicine, Kangbuk Samsung Hospital, Sungkyunkwan University School of Medicine, Seoul 03181, Korea; kcmd.sung@samsung.com; 6 Division of Gastroenterology and Hepatology, Department of Internal Medicine, Kangbuk Samsung Hospital, Sungkyunkwan University School of Medicine, Seoul 03181, Korea; choyk2004.cho@samsung.com; 7 Department of Occupational and Environmental Medicine, College of Medicine, Hanyang University, Seoul 04763, Korea; sjlee@hanyang.ac.kr; 8 Department of Family Medicine, Kangbuk Samsung Hospital, Sungkyunkwan University School of Medicine, Seoul 03181, Korea; 9 Usher Institute of Population Health Sciences and informatics, University of Edinburgh, Edinburgh EH8 9AG, UK; Sarah.Wild@ed.ac.uk; 10 Nutrition and Metabolism, Faculty of Medicine, University of Southampton, Southampton SO16 6YD, UK; C.D.Byrne@soton.ac.uk; 11 National Institute for Health Research Southampton Biomedical Research Centre, University Hospital Southampton, Southampton SO16 6YD, UK

**Keywords:** Nonalcoholic fatty liver disease, Hepatic fibrosis, Cardiovascular health metrics, cohort study

## Abstract

Cardiovascular Health (CVH) metrics scores are associated with cardiovascular disease but whether CVH scores are associated with nonalcoholic fatty liver disease (NAFLD) is uncertain. Our aim was to investigate associations between CVH scores and development or regression of NAFLD. A cohort study was performed in Korean adults who underwent a comprehensive health examination. The CVH metrics were defined according to the American Heart Association Life’s Simple 7 metrics, ranging from 0 (all metrics considered unhealthy) to 7 (all metrics considered healthy). Fatty liver was diagnosed by ultrasound, and liver fibrosis assessed using NAFLD fibrosis score (NFS). Among 93,500 participants without NAFLD or fibrosis at baseline, 15,899 developed NAFLD, and 998 developed NAFLD plus intermediate/high NFS. Healthy CVH metrics were inversely associated with NAFLD and also NAFLD with fibrosis. In time-dependent models after updating the CVH score and confounders as time-varying covariate, the multivariable-adjusted hazard ratio (95% confidence intervals) for incident NAFLD plus intermediate/high NFS participants with CVH metrics score 2, 3, 4, 5, or 6–7 to those with score 0–1 were 0.86 (0.59–1.25), 0.51 (0.36–0.73), 0.44 (0.31–0.62), 0.20 (0.14–0.29) and 0.09 (0.05–0.14), respectively. Regression of NAFLD occurred in 9742/37,517 participants who had NAFLD at baseline with positive association with CVH metrics. Higher CVH scores were significantly associated with both (a) decreased incidence of NAFLD, and (b) regression of existing NAFLD. Promoting adherence to ideal CVH metrics can be expected to reduce the burden of NAFLD as well as cardiovascular disease.

## 1. Introduction

Non-alcoholic fatty liver disease (NAFLD) is the most common chronic liver disease and includes a spectrum from simple steatosis to non-alcoholic steatohepatitis (NASH) with varying degrees of fibrosis and cirrhosis [1]. In addition to its liver-related complications, NAFLD is closely associated with obesity, hypertension, type 2 diabetes, hyperlipidemia, and insulin resistance, all of which are also risk factors for cardiovascular disease (CVD) [2]. Furthermore, NAFLD is considered hepatic manifestation of metabolic syndrome and has been associated with an increased risk for CVD and all-cause mortality [3,4]. There is no currently approved pharmacotherapy for NAFLD. Therefore, lifestyle changes are the first line of primary and secondary prevention of NAFLD and associated hepatic and extra-hepatic consequences.

In 2010, the American Heart Association (AHA) published recommendations for the general population to reduce cardiovascular morbidity and mortality by addressing seven specific cardiovascular health (CVH) behaviors and factors (Life’s Simple 7). The Life’s Simple 7 is a comprehensive and easily applicable assessment tool in clinical settings to promote adherence to healthy behaviors and comprise smoking, diet, physical activity, body mass index (BMI), blood pressure, total cholesterol, and fasting glucose [5]. While primary prevention is about treating risk factors to prevent CVD when risk factors arise, primordial prevention refers to avoiding the development of risk factors in the first place [5,6]. The Life’s Simple 7 has been introduced with the definition of ideal CVH metrics and an emphasis on the importance of primordial prevention of CVD through achievement and maintenance of ideal CVH metrics [5,7]. Previous studies, including a meta-analysis of prospective studies on ideal CVH metrics, have demonstrated a strong inverse association between ideal CVH metrics and both CVD and non-CVD outcomes, including diabetes mellitus, cancer, and all-cause mortality [8,9,10,11]. Although previous studies have shown associations between NAFLD and individual behavioral factors, such as diet and physical activity [12], to date it remains uncertain whether ideal CVH metrics are associated with development or regression of NAFLD.

We hypothesized that healthy CVH metrics would be associated with decreased development of incident NAFLD and fibrosis and increased regression of NAFLD. If targeting healthy numbers of CVH metrics (as a management/treatment strategy) was relevant to ameliorating NAFLD, focusing on improving CVH metrics might be a good public health strategy to reduce not only CVD but also NAFLD. Our aim was to investigate associations between CVH scores and: (a) development of incident NAFLD and (b) regression of NAFLD in subjects with existing NAFLD at baseline.

## 2. Materials and methods

### 2.1. Study Population

This study was part of the Kangbuk Samsung Health Study, a cohort study of Korean men and women 18 years or older who underwent annual or biennial health screening examinations at Kangbuk Samsung Hospital Total Healthcare Centers in Seoul and Suwon, South Korea as previously described [13]. The present analysis included all participants who underwent examinations, including abdominal ultrasonography (US) and had information on all 7 components of Life’s Simple 7 CVH metrics from March 1, 2011 (when food frequency questionnaires (FFQ) were added as part of the health questionnaires) to December 2016 (*n* = 188,834).

**Figure 1:**
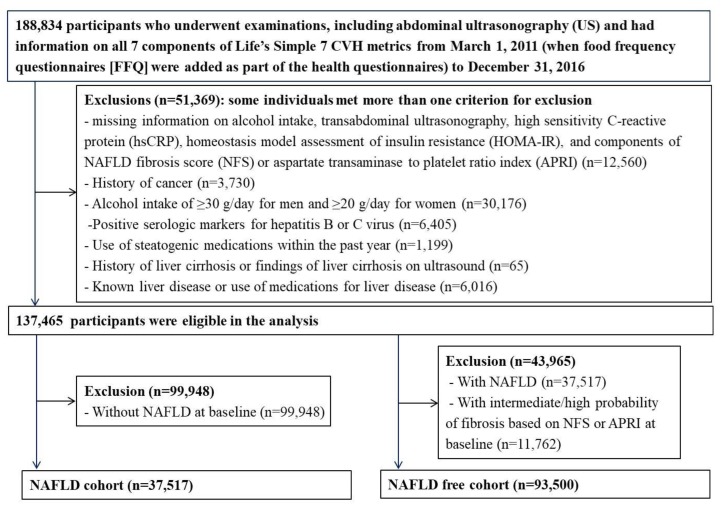
Flow diagram for the selection of study subjects. APRI, aspartate transaminase to platelet ratio index; FFQ, food frequency questionnaires; HOMA-IR, homeostasis model assessment of insulin resistance; hsCRP, high sensitivity C-reactive protein; NAFLD, non-alcoholic fatty liver disease; NFS, NAFLD fibrosis score; US, ultrasonography.

We excluded 51,369 participants at baseline for the following reasons: missing information on alcohol intake, transabdominal ultrasonography, high sensitivity C-reactive protein (hsCRP), homeostasis model assessment of insulin resistance (HOMA-IR), and components of NAFLD fibrosis score (NFS) or aspartate transaminase to platelet ratio index (APRI); alcohol intake ≥30 g/day for men and ≥20 g/day for women [1,14]; a history of cancer; known liver disease or use of medications for liver disease; history of liver cirrhosis or findings of liver cirrhosis on ultrasonography; positive serologic markers for hepatitis B or C virus; and use of steatogenic medications within the past year such as valproate, amiodarone, methotrexate, tamoxifen, or corticosteroids [1]. Because some participants met more than one exclusion criterion, 137,465 participants ultimately were eligible for this study (Figure 1).

Out of them, 37,517 (27.3%) were identified in the NAFLD cohort at the initial examination. Out of 99,948 subjects without NAFLD at baseline, we further excluded 11,762 subjects with intermediate/high probability of fibrosis based on NFS or APRI at baseline. The final study cohort for the analysis of associations between CVH scores and incident NAFLD and incident NAFLD with fibrosis therefore consisted of 93,500 subjects.

This study was approved by the Institutional Review Board of Kangbuk Samsung Hospital (IRB No. 2018-09-031), which waived the requirement for informed consent because de-identified retrospective data were used.

### 2.2. Measurements

Baseline and follow-up examinations were performed at Kangbuk Samsung Hospital Health Screening Center clinics. Standardized, self-administered questionnaires were used to obtain data on demographic characteristics, lifestyle factors, medical history, and medication use, as previously described [13].

Alcohol consumption was categorized as 0, ≤10, and >10 g/day. Physical activity level was evaluated using the validated Korean version of the International Physical Activity Questionnaire (IPAQ) short form [15]. A self-administered food frequency questionnaire (FFQ) using 103-items was used to evaluate usual dietary intake, which was designed and validated for use in Korea [16]. The FFQ consists of questions on the frequency and portion size of 103-items over the previous 12 months. A database of recipes, portion sizes, and nutrients was constructed using a food composition table from the Korean Nutrition Society. Height, weight and blood pressures (BP) were measured by trained nurses. Hypertension was defined as a systolic blood pressure ≥140 mmHg, a diastolic blood pressure ≥90 mmHg, or use of antihypertensive medications.

Information regarding reproductive factors was collected using self-administered, standardized questionnaires that asked about menopausal status, frequency and regularity of menstrual periods, and use of hormone replacement therapy and oral contraceptives. Parity was assessed from the number of reported live and still births. Menopause was defined as amenorrhea lasting for 12 or more months; in addition, women older than 55 years without information available regarding menopause were considered postmenopausal.

Fasting blood tests included total cholesterol, high-density lipoprotein-cholesterol (HDL-C), triglyceride (TG), alanine transaminase (ALT), glucose, and hsCRP. Insulin resistance was calculated using the homeostasis model (HOMA-IR) as follows: fasting insulin (mg/dL) × fasting glucose (mg/dL)/405.

### 2.3. Definition of CVH Metrics

The CVH metrics were defined according to the AHA Life Simple 7 factors [5]. Ideal CVH metrics were defined as follows: (1) Smoking: never or former smoker; (2) Physical activity: ≥75 min/week of vigorous intensity physical activity, ≥150 min/week of moderate or moderate plus vigorous intensity physical activity; (3) Diet: 4 or 5 healthy dietary components as defined below; (4) Total cholesterol <200 mg/dL; (5) BP < 120/80 mmHg; (6) Fasting glucose <100 mg/dL; (7) BMI < 23 kg/m^2^ based on the proposed cut-off for the diagnosis of overweight or obesity in Asians [17]. The diet scores were determined based on 5 healthy dietary components as follows: fruits and vegetables ≥450 g/day; fish ≥198 g/week; fiber-rich whole grain ≥85 g/day; sodium <1500 mg/day; sugar-sweetened beverages ≤1 L/week. An ideal diet score was defined as 4 or more healthy components. Korean meals usually comprise a bowl of cooked rice with a seasoned mixed soup and multiple side dishes. The median number of healthy diet components for CVH metrics in the subjects was 2 (interquartile range 1–2). The ratios of carbohydrate, protein, and fat to total energy intake were 67.4%, 13.9% and 18.7%, respectively, which approximately correspond to the recommended energy intake from carbohydrate 55%–65%, protein 7%–20% and fat 15%–30% according to the Dietary Reference Intakes for Koreans.

To calculate the CVH score, each ideal CVH component was awarded 1 point. The total ideal CVH score is defined as the sum of the values of each of the 7 ideal CVH metrics (range 0 to 7 points). The CVH scores were categorized as 0–1, 2, 3, 4, 5, and 6–7 because only 735 (0.53%) and 3,386 (2.46%) subjects had scores of 0 and 7 CVH, respectively. Therefore, in summary low CVH scores would be considered unhealthy and high CVH scores would be considered healthy.

### 2.4. Non-Alcoholic Fatty Liver Disease and Non-Invasive Fibrosis Indices

The diagnosis of fatty liver was made using abdominal ultrasound (US) by experienced radiologists who were blinded to the aim of the present study and was determined using standard criteria, including the presence of a diffuse increase in fine echoes in the liver parenchyma compared with kidney or spleen parenchyma, deep beam attenuation, and bright vessel walls [18]. Inter-observer and intra-observer reliability for the diagnosis of fatty liver were substantial (kappa statistic of 0.74) and excellent (kappa statistic of 0.94), respectively [13]. We defined NAFLD as the presence of a fatty liver on ultrasonography in the absence of excessive alcohol consumption (a threshold <20 g/day for women and <30 g/day for men) and other identifiable causes of hepatic steatosis (as described in the exclusion criteria) [1].

To assess the risk of severe NAFLD, the NFS was used. The NFS was calculated using the following formula: NFS = −1.675 + 0.037 × age (years) + 0.094 × BMI (kg/m^2^) + 1.13 × impaired fasting glycemia or diabetes (yes = 1, no = 0) + 0.99 × AST/ALT ratio − 0.013 × platelet (×109/L) − 0.66 × albumin (g/dL). Subjects were classified into three groups according to probability of advanced liver fibrosis: high (NFS > 0.676), intermediate (NFS: 0.676 to −1.455), and low (NFS < −1.455) [19]. In a sensitivity analysis, APRI was used and calculated according to the following formula: APRI = 100× (AST/upper limit of normal)/platelet count (×10^9^/L). Based on the probability of advanced fibrosis, subjects were also categorized as: high (APRI > 1.5), intermediate (APRI: 0.5 to 1.5), and low (APRI < 0.5).

### 2.5. Statistical Analyses

Descriptive statistics were used to summarize the participants’ characteristics according to the number of CVH metrics. To determine linear trends of incidence, the number of categories was used as a continuous variable and tested on each model.

We examined the association between CVH scores and the development and regression of NAFLD. The primary endpoint was development of NAFLD and the development of NAFLD with fibrosis based on NFS. Due to the very small number of participants who developed NAFLD with a high probability of advanced fibrosis (NFS > 0.676) during follow-up, we combined the intermediate and high fibrosis scores and used NAFLD with intermediate/high fibrosis scores (≥−1.455) as an endpoint. We also evaluated the association between ideal CVH metrics and NAFLD regression.

The follow-up duration for each participant extended from the baseline exam until development of the endpoint or the last health exam conducted prior to December 31, 2016, whichever came first. Incidence rates were calculated as the number of incident cases divided by person-years of follow-up. Since the development or regression of NAFLD was known to have developed between the two visits, but the precise time at which it occurred was not known, we used a parametric proportional hazard model to account for this type of interval censoring [20]. In these models, the baseline hazard function was parameterized with restricted cubic splines in log time with four degrees of freedom.

We used parametric proportional hazards models to estimate the hazard ratios (HRs) with 95% confidence intervals (CIs) for the development of incident NAFLD or regression of existing NAFLD. We initially adjusted for age and sex. Model 1 was further adjusted for study center (Seoul, Suwon), year of screening exam, alcohol intake, educational level, history of hypertension, and history of CVD. Model 2 was further adjusted for HOMA-IR and hsCRP. To evaluate the effects of changes in CVH scores and other covariates during the follow-up period, we performed additional analyses introducing the CVH scores and other factors as time-varying covariates in the models.

Statistical analyses were carried out using STATA version 15.0 (StataCorp LP, College Station, TX, USA). All *P* values < 0.05 were considered statistically significant.

## 3. Results

In 93,500 participants without NAFLD at baseline, the mean (standard deviation (SD)) age was 36.1 (6.4) years, and 42.8% of the participants were men. The proportion of those with a college education or higher was 86.5% (Table 1). The median CVH score was 3. In 37,517 participants with NAFLD (Table A1), the mean (SD) age was 39.2 (7.4) years, 83.7% of them were men. Increasing number of ideal CVH metrics met was associated with younger age; female sex; less smoking; lower alcohol intake; lower energy intake; lower BP; and lower levels of glucose, total cholesterol, LDL-C, triglycerides, ALT, hsCRP, and HOMA-IR. Participants with higher CVH scores were more likely to have higher physical activity and have higher HDL-C than were those with lower scores (Table 1 and Table A1).

Table 2 shows the development of NAFLD and NAFLD plus intermediate/high probability of advanced fibrosis among the NAFLD-free participants with low NFS at baseline (*n* = 93,500). During 328,760.7 person-years of follow-up, 15,899 participants developed NAFLD (incidence rate, 48.4/1,000 person-years). The median follow-up period for participants was 3.7 years (interquartile range, 2.0–4.8, maximum 6.7). Increasing CVH scores were significantly associated with a decreased risk for incident NAFLD in a dose-response relationship (*P* for trend <0.001). The multivariable-adjusted HR (95% CI) for development of NAFLD comparing participants with ideal CVH scores of 2, 3, 4, 5, or 6–7 to those with score of 0–1 were 0.88 (0.80–0.96), 0.74 (0.68–0.80), 0.56 (0.51–0.61), 0.39 (0.36–0.42) and 0.23 (0.21–0.25), respectively (Model 1). This association remained significant after further adjustment for HOMA-IR and hsCRP (Model 2). During 353,133.5 person-years of follow-up, 998 participants developed NAFLD plus intermediate/high NFS (incidence rate, 2.8/1,000 person-years). The multivariable-adjusted HR (95% CI) for development of NAFLD plus intermediate/high NFS when comparing participants with ideal CVH metrics score of 2, 3, 4, 5, or 6–7 to those with score of 0–1 were 0.88 (0.65–1.19), 0.67 (0.50–0.89), 0.56 (0.42–0.75), 0.29 (0.21–0.39) and 0.17 (0.11–0.24), respectively. When the association between CVH score and incident NAFLD was evaluated after updating the CVH score and confounders as time-varying covariates, the association between CVH score and development of NAFLD and NAFLD plus intermediate/high NFS was stronger in time-dependent models than it was in the original analysis. In the sensitivity analysis using APRI instead of NFS, we also found an inverse association between CVH score and NAFLD with fibrosis (Table 3).

Table 4 shows the regression of NAFLD according to CVH score in subjects with existing NAFLD at baseline (*n* = 37,517). During 128,481.5 person-years of follow-up, 9,742 participants showed regression of NAFLD (incidence rate, 75.8/1,000 person-years). The multivariable adjusted HR (95% CI) for NAFLD regression, comparing participants with CVH scores of 2, 3, 4, 5, or 6–7 to those with score 0–1 were 1.13 (1.04–1.23), 1.21 (1.12–1.31), 1.41 (1.30–1.53), 1.70 (1.56–1.86), and 2.28 (2.04–2.55), respectively (Model 1). These positive associations did not change after adjusting for HOMA-IR and the hsCRP level (Model 2). In the time-dependent analyses, the association between CVH score and NAFLD regression was stronger than in the original analysis. 

The association of CVH score with NAFLD development and regression was similarly observed in women after further adjustment for menopausal status and oral contraceptives (Table A2).

## 4. Discussion

In the cohort without NAFLD at baseline, there was an inverse independent association between the number of ideal CVH metrics and development of: (a) overall NAFLD and (b) NAFLD with fibrosis based on non-invasive measures of liver fibrosis indices. Furthermore, in subjects with existing NAFLD at baseline, CVH scores were independently associated with increased regression of NAFLD. These associations were pronounced when changes in ideal CVH metrics and confounders during follow-up were treated as time-varying covariates. Thus, our findings suggest that ideal CVH metrics has beneficial effects for both a decrease in the development of new-onset NAFLD and improvement of existing NAFLD over time.

As there are no licensed pharmaceutical treatments for NAFLD, a comprehensive approach targeted at lifestyle modification remains the cornerstone of clinical management of NAFLD [1,14]. Previous studies have demonstrated that lifestyle modifications are associated with decreased incidence of NAFLD development and progression [21,22,23,24]. These modifications include avoiding smoking, maintaining a normal weight, losing weight (7%–10%), eating a healthy diet (e.g., Mediterranean diet), and regular physical activity. The Mediterranean diet has been reported to be beneficial for prevention of multiple chronic diseases including NAFLD [25]. Both Mediterranean diet and healthy dietary component of CVH metrics are based on a food intake high in fruit and vegetables and fiber-rich whole grains while healthy dietary component of CVH metrics, specifically designed for improving cardiovascular health, includes a reduction in sodium intake and sugar-sweetened beverages [5]. However, most previous studies have focused on individual lifestyle factors in relation to NAFLD development [21,22]. Similarly, health factors (BP, glucose, and cholesterol) have been suggested to be associated with NAFLD development [14]. Multiple studies and meta-analyses have suggested the association of metabolic comorbidities (hypertension, type 2 diabetes, hyperlipidemia and metabolic syndrome) with NAFLD [26]. Although individual factors are well known traditional risk factors for CVD, the AHA has introduced is as a comprehensive and easily applicable assessment tool in clinical settings to promote adherence to healthy behaviors and ideal health factors, and not just to treat adverse risk factors when they arise. However, at present little is known about the association between healthy CVH metrics and development of, or regression of NAFLD. Very recently, a prospective cohort study of 3424 middle-aged and elderly Chinese participants reported an inverse association between number of ideal CVH metrics and development of NAFLD [27]. However, the study was limited by lack of dietary information and its use of only six components of Life’s Simple 7 metrics [27]. Our study’s large sample size, prospective design, repeated measures of all 7 CVH metrics components, ultrasonography and noninvasive fibrosis score, and availability of other confounders allowed us to evaluate the impact of ideal CVH metrics on both NAFLD development and regression. We found that higher CVH scores were associated with a decreased risk of: (a) developing NAFLD, and (b) NAFLD with fibrosis, as well as (c) increased regression of existing NAFLD. Our study extends the range of health outcomes associated with a beneficial role of ideal CVH metrics in NAFLD in addition to CVD, incident cancer, and all-cause mortality [11,28,29].

Our study has several limitations. First, abdominal ultrasonography was used to diagnose fatty liver instead of liver biopsy. However, ultrasonography is widely used in both clinical practice and epidemiology studies because of its acceptable accuracy in detecting fatty liver in a noninvasive nature [30]. Second, we used non-invasive fibrosis indices to diagnose liver fibrosis in NAFLD [19]. The NAFLD fibrosis score has been developed and validated in a heterogeneous group of NAFLD patients and correlates with advanced fibrosis confirmed by liver biopsy [19]. Third, the lifestyle variables were collected using self-administered structured questionnaires that are used in health checkup programs in Korea as part of the National Health Insurance plan. Therefore, measurement errors or misclassifications could contribute to some degree of residual confounding. Finally, our study population was relatively highly educated, young, and middle-aged Koreans, possibly limiting generalizability to other populations from other ethnic groups or with different age, demographic, and health behavior characteristics. On the other hand, our findings derived from a cohort of asymptomatic and relatively young people are less likely to have been affected by survivor bias, biases related to comorbidities, or those related to the use of multiple medications than are older cohorts.

In conclusion, a higher number of ideal CVH metrics was independently associated with decreased risk of incident NAFLD with and without liver fibrosis. Furthermore, higher number of ideal CVH metrics was associated with regression of existing NAFLD. Promoting adherence to ideal CVH metrics can provide a feasible and effective approach to prevent and treat NAFLD. This method may help reduce the burden of NAFLD as well as other chronic diseases including CVD.

## Figures and Tables

**Table 1:** Baseline characteristics by cardiovascular health metrics (CVH) scores among 93,500 participants without nonalcoholic fatty liver disease (NAFLD).

Characteristics	Overall	Number of Cardiovascular Health Metrics (CVH Scores)						*P* for Trend
		0–1	2	3	4	5	6–7	
Number of participants	93,500	1240	4186	9999	19,267	29,065	29,743	
Age (years)	36.1 (6.4)	37.9 (6.1)	37.6 (6.4)	37.4 (6.7)	36.7 (6.7)	35.9 (6.4)	35.0 (5.9)	<0.001
Men (%)	42.8	98.0	91.6	81.8	60.2	34.5	17.3	<0.001
Alcohol intake (%) ^a^	26.1	62.5	53.9	46.5	33.1	21.0	14.3	<0.001
Current smoker (%)	18.7	85.0	65.6	48.2	28.1	10.6	1.3	<0.001
HEPA (%)	14.9	1.9	5.4	8.3	10.4	11.6	25.2	<0.001
Education level (%) ^b^	86.5	85.7	87.8	88.2	86.0	86.1	86.4	0.002
Diabetes (%)	0.7	3.6	2.4	1.3	0.9	0.4	0.2	<0.001
Hypertension (%)	4.5	20.8	14.6	10.4	5.8	2.8	1.1	<0.001
History of CVD (%)	0.5	0.5	0.6	0.6	0.6	0.5	0.3	<0.001
Medication for hyperlipidemia (%)	0.7	1.4	1.2	1.4	1.0	0.6	0.3	<0.001
SBP	105.2 (11.9)	123.5 (9.9)	118.9 (11.2)	114.4 (11.6)	108.5 (11.4)	103.0 (10.2)	99.4 (8.7)	<0.001
DBP	67.1 (8.9)	80.4 (8.5)	76.5 (9.1)	73.2 (9.3)	69.1 (8.8)	65.6 (7.8)	63.3 (6.7)	<0.001
Glucose	91.3 (9.0)	103.2 (16.0)	98.5 (13.1)	95.4 (11.0)	92.8 (9.4)	90.2 (7.3)	88.6 (6.2)	<0.001
Total cholesterol	187.2 (31.5)	223.3 (27.8)	214.4 (30.1)	205.3 (31.7)	196.8 (32.0)	185.8 (30.1)	170.9 (21.7)	<0.001
LDL-C (mg/dL)	112.8 (29.2)	147.3 (26.2)	140.7 (28.2)	132.4 (29.4)	122.8 (28.9)	110.6 (26.5)	96.5 (20.2)	<0.001
HDL-C (mg/dL)	62.2 (14.6)	54.1 (12.0)	54.9 (12.9)	56.5 (13.6)	60.3 (14.8)	63.9 (14.9)	65.1 (13.5)	<0.001
Triglycerides (mg/dL)	75 (57–103)	134.5 (101–184)	118 (88–160)	102 (77–138)	86 (65–116)	71 (56–95)	62 (50–80)	<0.001
ALT (U/L)	15 (11–20)	24 (19–32)	22 (17–29)	19 (15–26)	17 (12–22)	14 (11–18)	13 (10–16)	<0.001
hsCRP (mg/L)	0.3 (0.2–0.6)	0.5 (0.3–1.0)	0.5 (0.3–0.9)	0.4 (0.3–0.9)	0.4 (0.2–0.7)	0.3 (0.2–0.6)	0.3 (0.2–0.5)	<0.001
HOMA-IR	1.00 (0.68–1.43)	1.44 (1.03–1.97)	1.30 (0.89–1.81)	1.18 (0.81–1.67)	1.07 (0.72–1.54)	0.97 (0.65–1.37)	0.89 (0.61–1.26)	<0.001
Total energy intake (kcal/day) ^c^	1509.6 (1155.7–1896.0)	1678.1 (1397.9–2053.6)	1665.1 (1370.4–2049.8)	1647.5 (1330.6–2023.7)	1582.6 (1240.7–1965.8)	1495.9 (1149.2–1878.4)	1387.7 (1029.7–1778.9)	<0.001

Data are mean (standard deviation), median (interquartile range), or percentage. ^a^ ≥10 g of ethanol per day. ^b^ ≥College graduate. ^c^ Among 95,031 participants with plausible estimated energy intake (within three standard deviations of the log-transformed mean energy intake). ALT, alanine aminotransferase; CAC, coronary artery calcification; HDL-C, high-density lipoprotein-cholesterol; HOMA-IR, homeostasis model assessment of insulin resistance; hsCRP, high sensitivity C-reactive protein.

**Table 2:** Development of NAFLD, intermediate/high probability of advanced fibrosis based on NFS, and NAFLD plus intermediate/high probability of advanced fibrosis based on NFS by cardiovascular health metrics at baseline among 93,500 NAFLD-free participants with a low probability of advanced fibrosis at baseline.

Number of Cardiovascular Health Metrics (CVH Scores)	PY	Incident Cases	Incidence Density (per 1000 PY)	Age- and Sex-Adjusted HR (95% CI)	Multivariable-Adjusted HR ^a^ (95% CI)		HR (95% CI) ^b^ in a Model Using Time-Dependent Variables
					Model 1	Model 2	
NAFLD							
0–1	4329.8	601	138.8	1.00 (reference)	1.00 (reference)	1.00 (reference)	1.00 (reference)
2	14468.3	1737	120.1	0.91 (0.83–1.00)	0.88 (0.80–0.96)	0.91 (0.83–0.99)	0.89 (0.80–0.99)
3	34781.4	3369	96.9	0.79 (0.72–0.86)	0.74 (0.68–0.80)	0.76 (0.70–0.83)	0.68 (0.61–0.75)
4	67564.9	4408	65.2	0.62 (0.57–0.67)	0.56 (0.51–0.61)	0.60 (0.55–0.66)	0.52 (0.47–0.57)
5	102796.4	3824	37.2	0.44 (0.40–0.48)	0.39 (0.36–0.42)	0.43 (0.39–0.47)	0.32 (0.29–0.36)
6–7	104819.9	1960	18.7	0.26 (0.24–0.29)	0.23 (0.21–0.25)	0.26 (0.23–0.28)	0.17 (0.15–0.19)
*P* for trend				<0.001	<0.001	<0.001	<0.001
NAFLD + Intermediate/high based on NFS							
0–1	5499.5	58	10.5	1.00 (reference)	1.00 (reference)	1.00 (reference)	1.02 (reference)
2	17594.0	153	8.7	0.86 (0.64–1.16)	0.88 (0.65–1.19)	0.91 (0.67–1.24)	0.86 (0.59–1.25)
3	40496.0	243	6.0	0.65 (0.49–0.86)	0.67 (0.50–0.89)	0.70 (0.52–0.93)	0.51 (0.36–0.73)
4	74083.1	305	4.1	0.54 (0.41–0.72)	0.56 (0.42–0.75)	0.61 (0.46–0.81)	0.44 (0.31–0.62)
5	108107.9	168	1.6	0.27 (0.20–0.37)	0.29 (0.21–0.39)	0.32 (0.24–0.44)	0.20 (0.14–0.29)
6–7	107353.0	71	0.7	0.16 (0.11–0.22)	0.17 (0.11–0.24)	0.19 (0.13–0.27)	0.09 (0.05–0.14)
*P* for trend				<0.001	<0.001	<0.001	<0.001

^a^ Estimated from parametric proportional hazard models. Multivariable model 1 was adjusted for age, sex, center, year of screening exam, alcohol intake, education level, history of diabetes, history of hypertension, and history of cardiovascular disease; model 2: model 1 plus adjustment for HOMA-IR and hsCRP. ^b^ Estimated from parametric proportional hazard models with alcohol intake and number of cardiovascular health metrics as time-dependent categorical variables and baseline age, sex, center, year of screening exam, education level, history of diabetes, history of hypertension, and history of cardiovascular disease as time-fixed variables. BMI, body mass index; CI, confidence interval; CVH, cardiovascular health; HR, hazards ratio; NAFLD, nonalcoholic fatty liver disease; NFS, NAFLD fibrosis score; PY, person-year.

**Table 3:** Development of intermediate/high probability of advanced fibrosis based on APRI, and NAFLD plus intermediate/high probability of advanced fibrosis based on APRI by cardiovascular health metrics at baseline among 93,500 NAFLD-free participants with a low probability of advanced fibrosis at baseline.

Number of Cardiovascular Health Metrics (CVH Scores)	PY	Incident Cases	Incidence Density (per 1000 PY)	Age- and Sex-Adjusted HR (95% CI)	Multivariable-Adjusted HR^a^ (95% CI)		HR (95% CI) ^b^ in a Model Using Time-Dependent Variables
					Model 1	Model 2	
NAFLD + Intermediate/high based on APRI							
0–1	5600.0	22	3.9	1.00 (reference)	1.00 (reference)	1.00 (reference)	1.00 (reference)
2	17752.0	81	4.6	1.27 (0.79–2.03)	1.30 (0.81–2.09)	1.36 (0.85–2.19)	0.97 (0.55–1.69)
3	40718.9	116	2.8	0.86 (0.55–1.36)	0.90 (0.57–1.42)	0.95 (0.60–1.50)	0.67 (0.39–1.15)
4	74417.4	120	1.6	0.58 (0.37–0.92)	0.61 (0.39–0.97)	0.68 (0.42–1.07)	0.43 (0.25–0.74)
5	108238.4	111	1.0	0.46 (0.29–0.74)	0.50 (0.31–0.80)	0.56 (0.35–0.91)	0.32 (0.18–0.56)
6–7	107408.8	44	0.4	0.22 (0.13–0.38)	0.24 (0.14–0.41)	0.28 (0.16–0.47)	0.09 (0.04–0.17)
*P* for trend				<0.001	<0.001	<0.001	<0.001

^a^ Estimated from parametric proportional hazard models. Multivariable model 1 was adjusted for age, sex, center, year of screening exam, alcohol intake, education level, history of diabetes, history of hypertension, and history of cardiovascular disease; model 2: model 1 plus adjustment for HOMA-IR and hsCRP. ^b^ Estimated from parametric proportional hazard models with alcohol intake and number of cardiovascular health metrics as time-dependent categorical variables and baseline age, sex, center, year of screening exam, education level, history of diabetes, history of hypertension, and history of cardiovascular disease as time-fixed variables. BMI, body mass index; CI, confidence interval; CVH, cardiovascular health; HR, hazards ratio; NAFLD, nonalcoholic fatty liver disease; PY, person-year.

**Table 4:** Regression of NAFLD by cardiovascular health metrics at baseline among 37,517 participants with NAFLD.

Number of Cardiovascular Health Metrics (CVH Scores)	PY	Incident Cases	Incidence Density (per 1000 PY)	Age- and Sex-Adjusted HR (95% CI)	Multivariable-Adjusted HR^a^ (95% CI)		HR (95% CI) ^b^ in a Model Using Time-Dependent Variables
					Model 1	Model 2	
0–1	14541.8	806	55.4	1.00 (reference)	1.00 (reference)	1.00 (reference)	1.00 (reference)
2	27180.7	1716	63.1	1.11 (1.02–1.21)	1.13 (1.04–1.23)	1.05 (0.97–1.14)	1.00 (0.90–1.11)
3	35923.4	2466	68.6	1.19 (1.10–1.29)	1.21 (1.12–1.31)	1.06 (0.98–1.15)	1.13 (1.03–1.24)
4	31197.3	2558	82.0	1.37 (1.27–1.49)	1.41 (1.30–1.53)	1.16 (1.07–1.26)	1.35 (1.23–1.48)
5	15538.6	1605	103.3	1.65 (1.52–1.80)	1.70 (1.56–1.86)	1.32 (1.20–1.44)	1.63 (1.47–1.80)
6–7	4099.7	591	144.2	2.23 (2.00–2.49)	2.28 (2.04–2.55)	1.64 (1.46–1.83)	2.33 (2.07–2.63)
*P* for trend				<0.001	<0.001	<0.001	<0.001

^a^ Estimated from parametric proportional hazard models. Multivariable model 1was adjusted for age, sex, center, year of screening exam, alcohol intake, education level, history of diabetes, history of hypertension, and history of cardiovascular disease; model 2: model 1 plus adjustment for HOMA-IR and hsCRP. ^b^ Estimated from parametric proportional hazard models with alcohol intake and number of cardiovascular health metrics as time-dependent categorical variables and baseline age, sex, center, year of screening exam, education level, history of diabetes, history of hypertension, and history of cardiovascular disease as time-fixed variables. BMI, body mass index; CI, confidence interval; CVH, cardiovascular health; HR, hazards ratio; NAFLD, nonalcoholic fatty liver disease; PY, person-year.

## Appendix A

**Table A1:** Baseline characteristics by cardiovascular health metrics among 37,517 participants with nonalcoholic fatty liver disease (NAFLD).

Characteristics	Overall	Number of Cardiovascular Health Metrics (CVH Scores)						*P* for Trend
		0–1	2	3	4	5	6–7	
Number of participants	37,517	3795	7514	10,337	9448	5005	1418	
Age (years)	39.2 (7.4)	39.5 (6.3)	39.5 (7.1)	39.3 (7.4)	39.1 (7.7)	38.7 (7.8)	37.9 (7.7)	<0.001
Men (%)	83.7	97.2	92.7	88.2	79.4	67.3	54.2	<0.001
Alcohol intake ^a^ (%)	42.1	58.8	50.3	44.7	36.3	28.5	22.4	<0.001
Current smoker (%)	36.4	79.3	55.7	38.3	21.8	8.2	1.6	<0.001
HEPA (%)	14.3	2.1	6.1	11.2	17.5	26.8	46.3	<0.001
Education level ^b^ (%)	87.3	87.3	88.1	87.4	87.3	85.3	88.4	0.019
Diabetes (%)	7.1	14.3	10.3	7.3	4.9	2.4	1.2	<0.001
Hypertension (%)	17.0	29.4	23.5	17.8	12.9	7.4	4.8	<0.001
History of CVD (%)	1.0	0.8	1.0	1.0	1.2	0.9	0.8	0.720
Lipid lowering drug (%)	3.4	2.6	3.3	3.8	3.8	3.1	2.7	0.301
SBP	115.4 (12.1)	125.0 (10.6)	120.6 (11.7)	116.2 (11.5)	112.0 (10.5)	108.1 (9.4)	104.4 (8.9)	<0.001
DBP	74.2 (9.7)	81.7 (8.8)	78.3 (9.5)	74.8 (9.2)	71.5 (8.3)	68.6 (7.3)	66.2 (6.9)	<0.001
Glucose	99.4 (17.6)	110.2 (25.8)	103.8 (20.4)	99.5 (16.4)	96.1 (13.4)	93.1 (9.5)	91.1 (6.4)	<0.001
Total cholesterol	205.9 (35.6)	229.3 (31.8)	219.5 (32.6)	209.0 (34.8)	197.8 (33.8)	185.5 (28.4)	175.1 (22.7)	<0.001
LDL-C (mg/dL)	135.2 (32.0)	153.2 (28.6)	146.2 (30.0)	138.2 (31.9)	129.1 (30.7)	118.1 (26.1)	107.4 (22.2)	<0.001
HDL-C (mg/dL)	48.5 (11.0)	46.5 (9.9)	47.4 (10.2)	48.1 (10.6)	48.9 (11.2)	50.4 (11.9)	53.2 (12.9)	<0.001
Triglycerides (mg/dL)	136 (98–189)	179 (134–246)	158 (118–213)	141 (104–193)	123 (91–171)	106 (78–147)	90 (67–124)	<0.001
ALT (U/L)	29 (20–44)	38 (27–56)	34 (24–51)	31 (22–46)	27 (19–40)	23 (16–34)	19 (14–29)	<0.001
hsCRP (mg/L)	0.7 (0.4–1.4)	0.9 (0.5–1.8)	0.8 (0.5–1.5)	0.8 (0.4–1.4)	0.7 (0.4–1.4)	0.6 (0.3–1.2)	0.5 (0.3–0.9)	<0.001
HOMA–IR	1.74 (1.20–2.51)	2.24 (1.58–3.26)	1.99 (1.39–2.85)	1.80 (1.25–2.55)	1.61 (1.11–2.29)	1.43 (1.00–1.99)	1.21 (0.85–1.73)	<0.001
Total energy intake (kcal/day) ^c^	1643.7 (1307.3–2046.2)	1704.2 (1414.6–2101.5)	1699.2 (1373.9–2102.6)	1652.3 (1325.7–2055.9)	1616.2 (1268.3–2011.0)	1567.3 (1213.6–1986.8)	1515.9 (1161.2–1959.8)	<0.001

Data are mean (standard deviation), median (interquartile range), or percentage. ^a^ ≥ 10 g of ethanol per day ^b^ ≥ college graduate ^c^ Among 34,779 participants with plausible estimated energy intake (within three standard deviations from the log-transformed mean energy intake). ALT, alanine aminotransferase; CAC, coronary artery calcification; HDL-C, high-density lipoprotein-cholesterol; hsCRP, high sensitivity C-reactive protein; HOMA-IR, homeostasis model assessment of insulin resistance.

**Table A2:** Hazard ratios (95% CI) for Development of NAFLD, intermediate/high probability of advanced fibrosis based on NFS and regression of NAFLD by cardiovascular health metrics at baseline among women.

Number of Cardiovascular Health Metrics (CVH Scores)	Multivariable-Adjusted HR ^a^ (95% CI)		
	For Development of NAFLD	For Progression of Intermediate/High Probability of Advanced Fibrosis Based on NFS	For Regression of NAFLD
0–1	1.00 (reference)	1.00 (reference)	1.00 (reference)
2	1.18 (0.71–1.96)	0.65 (0.27–1.55)	0.86 (0.57–1.30)
3	0.90 (0.55–1.46)	0.50 (0.22–1.11)	0.95 (0.65–1.41)
4	0.64 (0.39–1.04)	0.33 (0.15–0.73)	1.03 (0.71–1.52)
5	0.38 (0.24–0.63)	0.13 (0.06–0.29)	1.20 (0.82–1.77)
6–7	0.21 0.13–0.34)	0.08 0.03–0.18)	1.47 (0.99–2.18)
*P* for trend	<0.001	<0.001	<0.001

^a^ Estimated from parametric proportional hazard models. Multivariable model 1was adjusted for age, sex, center, year of screening exam, alcohol intake, education level, history of diabetes, history of hypertension, and history of cardiovascular disease, HOMA-IR, hsCRP, menopause, parity and oral contraceptives. BMI, body mass index; CI, confidence interval; CVH, cardiovascular health; HR, hazards ratio; NAFLD, nonalcoholic fatty liver disease; PY, person-year.
